# A Hybrid Crack Detection Approach for Scanning Electron Microscope Image Using Deep Learning Method

**DOI:** 10.1155/2021/5558668

**Published:** 2021-08-09

**Authors:** Lun Zhao, Yunlong Pan, Sen Wang, Liang Zhang, Md Shafiqul Islam

**Affiliations:** ^1^Institute of Intelligent Manufacturing Technology, Shenzhen Polytechnic, Shenzhen 518055, China; ^2^Shenzhen Institutes of Advanced Technology, Chinese Academy of Sciences, Shenzhen 518055, China; ^3^Faculty of Mechanical and Electrical Engineering, Kunming University of Science and Technology, Kunming 650500, China; ^4^Department of Mechanical Engineering, Faculty of Engineering, Blekinge Institute of Technology, 37179 Karlskrona, Sweden

## Abstract

The scanning electron microscope (SEM) is widely used in the analysis and research of materials, including fracture analysis, microstructure morphology, and nanomaterial analysis. With the rapid development of materials science and computer vision technology, the level of detection technology is constantly improving. In this paper, the deep learning method is used to intelligently identify microcracks in the microscopic morphology of SEM image. A deep learning model based on image level is selected to reduce the interference of other complex microscopic topography, and a detection method with dense continuous bounding boxes suitable for SEM images is proposed. The dense and continuous bounding boxes were used to obtain the local features of the cracks and rotating the bounding boxes to reduce the feature differences between the bounding boxes. Finally, the bounding boxes with filled regression were used to highlight the microcrack detection effect. The results show that the detection accuracy of our approach reached 71.12%, and the highest mIOU reached 64.13%. Also, microcracks in different magnifications and in different backgrounds were detected successfully.

## 1. Introduction

The scanning electron microscope (SEM) is a large precision instrument used for high-resolution microarea analysis. It has the characteristics of large depth of field, high resolution, intuitive imaging, strong stereo perception, and wide magnification range, and the sample to be tested can be rotated and tilted in a three-dimensional space [1–4]. In addition, it has the advantages of rich types of measurable samples, almost no damage and pollution to the original sample, and simultaneous acquisition of morphology, structure, composition, and crystallographic information, etc. [5–8]. At present, SEM has been widely used in the microscopic research of materials science, such as fracture analysis, microstructure morphology, and nanomaterial analysis. However, it will be a challenge to locate microcracks intuitively, quickly, and accurately in complex microtopography or microcracks with different sizes, shapes, and directions. Artificial intelligence algorithms have been widely used in various fields with the development of computer hardware equipment, and it will open up a new path of exploration for the microinvestigation of materials science.

The conventional method manually changes the magnification of the SEM image to find the region of interest in the microscopic morphology [9]. Although it is simple to operate, it is quite inefficient. Many scholars combined imaging technology with SEM images to find target areas of microstructure [10–13], or improved research efficiency by highlighting the feature of research object [14, 15]. However, imaging technology is greatly influenced by a research object. Researchers began to apply them to microstructure analysis, due to deep learning and machine learning having the ability to automatically extract useful information [16]. For example, five types of machine learning methods and one deep learning model were applied, respectively, to achieve the pixel-level mineral classification of SEM-EDS images [17]. Deep learning was used to remove SEM image noise and improved the measurement accuracy of line edge roughness [18]. U-Net [19] was used to analyze SEM images of mineral characterization, which distinguished effectively the mixed matrix mineral particles and organic clay aggregates [20]. Deep learning was applied to detect nanoparticles in microscopic images and uses Hough algorithm to detect particle edges [21]. Owing to the microscopic morphology of the fracture, its vicinity is quite complex and has a certain similarity with fatigue cracks. Pixel-based detection methods such as edge extraction or image segmentation are prone to misidentification. Therefore, considering used image-based object detection algorithms to predict microcracks in the micromorphology.

Deep learning is derived from machine learning and has outstanding performance in computer vision and natural language processing [22]. In the field of computer vision, the intelligent recognition of structures mainly includes image classification [23], object detection [24], and image segmentation [25, 26]. At present, many scholars have done a lot of work in the intelligent identification of surface cracks on structures. On the one hand, based on the YOLOv4 [27] algorithm, real-time detection of bridge cracks was achieved through network lightweight [28]. However, this method can obtain the overall feature of cracks at one time, which is not conducive to microscopic scenes with more complex backgrounds. The crack is divided into several similar crack patches to achieve weakly supervised semantic segmentation of cracks, which explained the feature of crack patches and crack have a certain similar [29]. The block-based detection method could tolerate noise, but the edge location was not accurate [30]. A hybrid detection method based on faster R-CNN [31], which used the Bayesian integration algorithm to optimize crack patches to suppress false detection [32]. On the other hand, through training support vector machines, the microstructure was successfully classified into 7 groups, indicating that computer vision technology can be applied to microscopic research [33]. An acoustic crack detection method for carbonized anvils was developed using deep learning [34]. A convolutional neural network was used to improve the arc welding robot to realize the online defect detection of aluminum alloy surface arc welding [35]. The DSOD [36] algorithm can successfully identify the initiation location of a fatigue crack in an alloy compound [37].

At present, deep learning algorithms are applied in the investigation of macrocracks has achieved phased results, but there are relatively few studies on microcracks in micromorphology. In order to further explore the intelligent detection of microcracks in the microtopography, this paper employs a dense rotating bounding box based on the deep learning model to intelligently detect microcracks in the microtopography. SEM images are grayscale images; the brightness and contrast information is diverse due to the influence of imaging technology. Inspired by the literature [29, 30, 32], we consider using the same bounding box to intensively and continuously label along the fatigue trajectory of the crack and adding the angle constraint of the bounding box to improve the positioning accuracy of the bounding box. Bounding boxes with rotation angles were first used in text detection algorithms such as EAST [38] and R2CNN [39]. Then, it was widely used in remote sensing image detection, such as RR-CNN [40] and R3Det [41], which performed well in sensing detection of ship remote. Using segmentation tasks in the bounding box of regression to predict the bounding box of rotation [42] and the single-stage detector of rotation based on RetinaNet [43] [44], both need to pass a postprocessing process of NMS [45]. Because the crack patches are arranged densely and continuously, it is easy to lose certain effective bounding boxes with relatively low score probabilities in the postprocessing process. Therefore, this article uses CenterNet [46], an anchor-free detector. The rotating frame prediction method is learned from literature [47]. First, CenterNet determines the target center point and then uses the angle information to calculate the intersection loss and realize the regression of the rotating bounding box. Since the result of target detection is to assign a predefined category label to detect all objects [48]. Each bounding box in this paper only represents the local area of the crack. Thus, inspired by the results of image segmentation, the regression shape of each bounding box was modified and the box was filled. The results show that the highest detection accuracy of the approach reaches 71.12%, and the highest mIOU reaches 64.13%. It not only reduces the regression error of the size and position of the bounding box but also can be applied to SEM images with different magnifications and backgrounds.

## 2. Methodology

### 2.1. Overview

**Figure 1:**
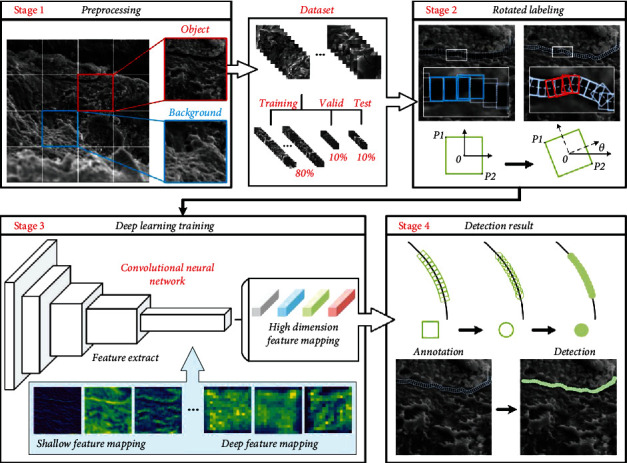
Illustration of our methods. Boxes are used in the cutting process, and the size is determined by the size of the image crack in stage 1. The white box is a partial enlargement of the labeled bounding box in stage 2. Only the images of the training dataset are used for training, and the images of the valid dataset are used to verify the current training effect during the training process in stage 3. Bounding boxes of different shapes are all derived from the same high-dimensional feature map in stage 4.

The workflow of our approach is shown in Figure 1. The original images through the same box were cut and initially filtered the object area and background area in stage 1. The background area will be abandoned, and the object area will be used for data augmentation to get the dataset. As shown by the red bounding boxes, a rotating bounding box was used to mark densely and continuously the crack target to obtain the data, which the neural network needs to learn and train in stage 2. The label box contains the local features of the crack. Crack trajectories of any shape were combined by using the deflection angle, which helps to reduce the difference in the shape of cracks between different bounding boxes. The marked data will be sent to the convolutional neural network for training in stage 3. During the training process, the feature extractor of the deep learning model will extract effective crack features and aggregate them into high-dimensional feature maps. Our deep learning network model is mainly composed of residual network [28] and CenterNet algorithm. Among them, the change of the feature map is shown in the blue filled box. The shallow feature map contains rich crack morphology and edge information, while the high-dimensional feature map contains rich semantic information, which expresses some key crack feature pixels in the image. We will get four high-dimensional feature maps when the training is finished, which are used to return the position, offset, size, and angle of the target bounding box. The regressed bounding box was used to predict the critical area of the crack and then modifies the regression form of the bounding box to get the final detection result, as shown in stage 4. In the following sections, the rotation annotation method, the regression method of the deep learning network model, and the bounding box will be introduced in Section 2.2 and Section 2.3.

### 2.2. Rotated Labeling

**Figure 2:**
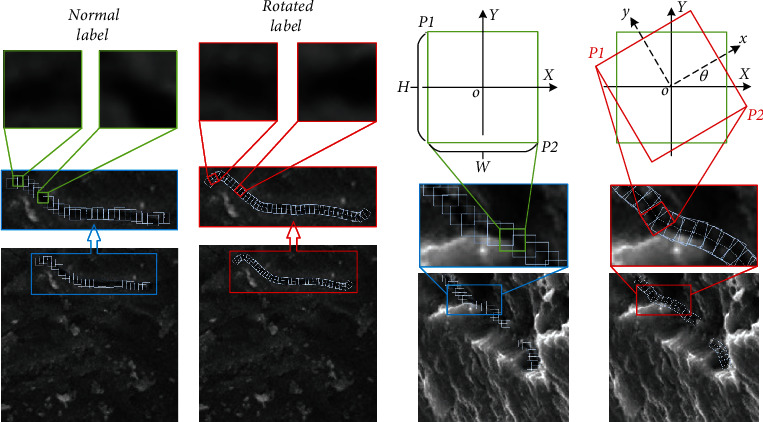
Comparison of different labeling. The center point of the rotating bounding box falls in the middle of the crack patch, and the *y*-axis is parallel to the crack propagation direction.

The direction of fatigue crack growth is mostly presented as a complex curve, due to specimens being affected by load form, alternating stress, surface state, chemical composition, and inclusions. Each bounding box corresponds to a complete detection target in traditional target detection methods. Limited by the diversity of crack shapes, it is not conducive to the extraction of effective features if a bounding box is used to express the characteristic information of the crack as a whole. Therefore, the bounding boxes express only the local area of the crack and express the complete crack information through dense and continuous labeling. Each label box contains the local information of the crack trajectory, but there are different degrees of difference in the information. It is found that the crack information in the conventional bounding box labeling may become irregular line segments, by comparing Figures 2(a) and 2(b). It can be a straight line perpendicular to a certain coordinate axis, or a curve with any angle. It will help the network model to extract more uniform crack features if we reduce the difference between different bounding boxes. Therefore, a rotating bounding box is proposed to replace the conventional bounding box. As shown in Figure 2(c), the conventional label box records the real position, size, and other information of the object, which is the content that the network model needs to learn and predict. As shown in Figure 2(d), the rotating bounding box adds the angle information of the target on the basis of the conventional bounding box. Densely arranged bounding boxes can effectively reduce irrelevant background information. On the basis, by adding angle information, the bounding boxes can be closer to the crack trajectory. Therefore, it can not only be combined into any form of crack trajectory but also can reduce the characteristic difference of crack information between different bounding boxes.

### 2.3. Detection Approach

#### 2.3.1. Network Model

**Figure 3:**
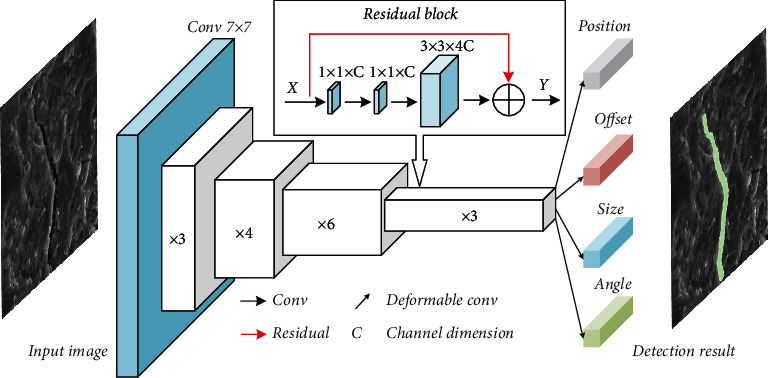
The structure of the deep learning model. All different scales use the same residual block structure, but the numbers used are 3, 4, 6, and 3.

The neural network model is a feature extractor used to extract key information of an image in the deep learning model. This paper uses ResNet50 [28] as the feature extractor. The network structure is shown in Figure 3 and Table 1. Firstly, a 7 × 7 convolution kernel is used to extract the rough information of the shallow network. Secondly, the features of the image at four different scales are extracted through the residual module, and a high-dimensional feature map with a width of 16 × 16 is obtained. Finally, the center point position, offset, bounding box size, and deflection angle are predicted by deformable convolution [29]. Among them, the residual module is composed of three convolutional layers and a residual connection. The output *Y* can be calculated as *X* + *F*(*X*) using the residual connection, which means that the output *Y* can learn the features from the input *X* [28].

**Figure 4:**
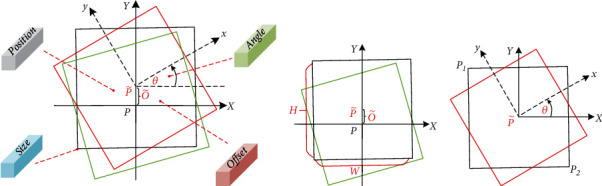
Illustration of rotated bounding box. The green box is ground true, and the red box is the test result. The solid line coordinate axis is the absolute coordinate based on ground true, and the dashed line coordinate axis is the relative coordinate based on the rotating bounding box.

Prediction in Table 1 is a process of using high-dimensional feature mapping to regress the rotating bounding box. As shown in Figure 4(a), four high-dimensional feature maps are used to predict the position, offset, bounding box size, and rotation angle of center point. The known center point position *P* and the predicted offset $\tilde{O}$ were used to determine the predicted center point coordinates $\tilde{P}$. Then, we return to the size of the bounding box to obtain the black bounding box, as shown in Figure 4(b). Finally, we rotate counterclockwise *θ* based on the same center point to get the prediction result shown in the red box in Figure 4(c). There is a training loss between the prediction result and the real bounding box, which is calculated as follows:

(1) $$\begin{array}{c} L_{\text{Position}}=\frac{1}{N}\underset{P}{\sum }\;{\left(1-{\overset{\wedge }{Y}}_{P}\right)}^{\alpha }\log\left({\hat{Y}}_{P}\right), \end{array}$$

(2) $$\begin{array}{c} L_{\text{Offset}}=\lambda_{S}\left(\frac{1}{N}\underset{P}{\sum }\;\left|\tilde{O}-\left(P-\tilde{P}\right)\right|\right), \end{array}$$

(3) $$\begin{array}{c} L_{\text{Size}}=\lambda_{O}\left(\frac{1}{N}\overset{N}{\underset{k=1}{\sum }}\;\left|{\tilde{S}}_{P}-S\right|\right), \end{array}$$

(4) $$\begin{array}{c} L_{\text{Angle}}=\left\{\begin{array}{cc} 0.5\theta^{2} & \text{if}\left|\theta \right|<1,\\ \left|X\right|-0.5 & \text{otherwise}, \end{array}\right. \end{array}$$

(5) $$\begin{array}{c} L_{\text{Loss}}=L_{\text{Position}}+L_{\text{Offset}}+L_{\text{Size}}+L_{\text{Angle}}. \end{array}$$

Among them, *P* is the true center point and $\tilde{P}$ is the predicted center point. $\hat{Y}\in {\left[0,1\right]}^{\left(W/4\right)\times \left(H/4\right)}$ is used to judge whether the target exists when $\hat{Y}=1$ indicates that the target exists. $\tilde{O}$ is the offset of center point, ${\tilde{S}}_{P}-S$ is the loss of the width and height of a single bounding box, and *λ*_*S*_ and *λ*_*O*_ are hyperparameters. *θ* is is the rotation angle of the bounding box, and the value range is [0, *π*].

#### 2.3.2. Detection as Circle

**Figure 5:**
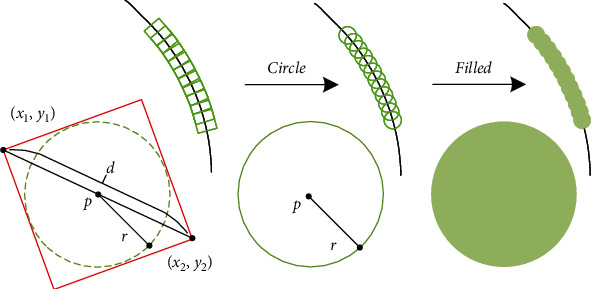
Illustration of filled regression. (*x*_1_, *x*_2_) and (*x*_3_, *x*_4_) data of each bounding box are obtained from the high-dimensional feature map. These data can be transformed into the center point *p* and the radius *r*, thereby changing the regression shape of the bounding box.

Most of crack colors in the SEM image are black or gray-black. The densely arranged bounding box is not conducive for people to observe the detection result if the predicted bounding box is directly returned to the crack. This paper proposes a method to modify the regression bounding box to further highlight the detection results in the SEM image. As shown in Figure 5, the center point of the prediction result can be calculated by (*x*_1_, *y*_1_) and (*x*_2_, *y*_2_). *d* is the length of the diagonal of the bounding box, calculated by $\sqrt{{\left(x_{1}-x_{2}\right)}^{2}+{\left(y_{1}-y_{2}\right)}^{2}}$. The green dashed circle in the red box is the inscribed circle of the bounding box, and its radius $r=d/\left(2\sqrt{2}\right)$. Therefore, the center and radius of the inscribed circle of the bounding box are determined. We only need to modify the detection code to realize the regression and filling of the circular bounding box. A curve that coincides with the crack track can be obtained when dense and continuous regression bounding boxes are combined.

## 3. Experiment

### 3.1. Implementation Details

**Figure 6:**
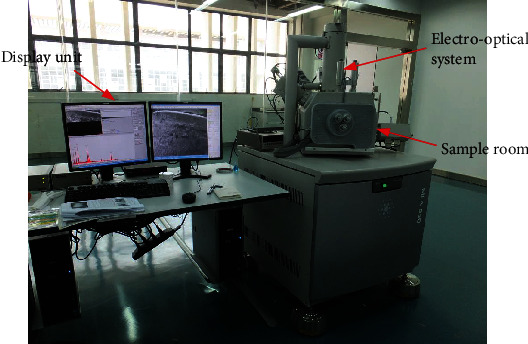
High vacuum scanning electron microscope.

The experimental equipment includes desktop computers (an Intel(R) Core(TM) i7-9700 CPU @ 3.00GHz,GPU NVIDIA GeForce RTX 2080S, 16GB of RAM, and Windows10-64 bit) and the high-vacuum scanning electron microscope produced by American FEI company, as shown in Figure 6. 20 images obtained from SEM on the fracture surface of the titanium alloy sample, the crack width in the images from 1 *μ*m to 20 *μ*m, and all the cracks caused by fatigue experiments. After data expansion processing, such as cropping and rotating the cracked area, 120 images are obtained. The interpolation algorithm was used to adjust the size of all images to adjust the size of all images to 512 × 512 at first. Then, we randomly selected 80 pictures as the training dataset, and 20 pictures as the verification dataset and test dataset. Our algorithm was run on the PyTorch deep learning framework with CUDA10.0 and cudnn7.6.4. All experiments use an iterative training strategy and Adam optimizer and set the total number of iterations to 5000. We set the initial learning rate to 0.8*e* − 4 and attenuate the learning rate by ten times after 3200 and 4200 iterations.

### 3.2. Evaluation Metrics

Accuracy (*P*), average intersection ratio (mIOU), *F*1 score, and average angle loss (mAng) will be used as the evaluation indicators of model performance in this paper, calculated as follows:

(6) $$\begin{array}{c} \text{Precision}=\frac{\text{TP}}{\text{TP}+\text{FP}}, \end{array}$$

(7) $$\begin{array}{c} \text{mAng}=\frac{1}{N}\sum \;\frac{\left|\theta -\tilde{\theta }\right|}{\pi }, \end{array}$$

(8) $$\begin{array}{c} \text{mIOU}=\frac{1}{N}\sum \;\frac{A⋂\;B}{A⋃\;B}. \end{array}$$

Among them, TP is a positive sample with correct prediction, FP is a positive sample with incorrect prediction, and FN is a negative sample with incorrect prediction. Precision refers to the proportion of positive samples in the prediction result, and mAng represents the average error between the predicted angle and the true angle. *A* is the real bounding box area, *B* is the predicted bounding box area, and mIOU represents the average intersection ratio between the predicted bounding box and the real bounding box.

### 3.3. Experimental Analysis and Result

#### 3.3.1. Comparison of Different Bounding Boxes

**Figure 7:**
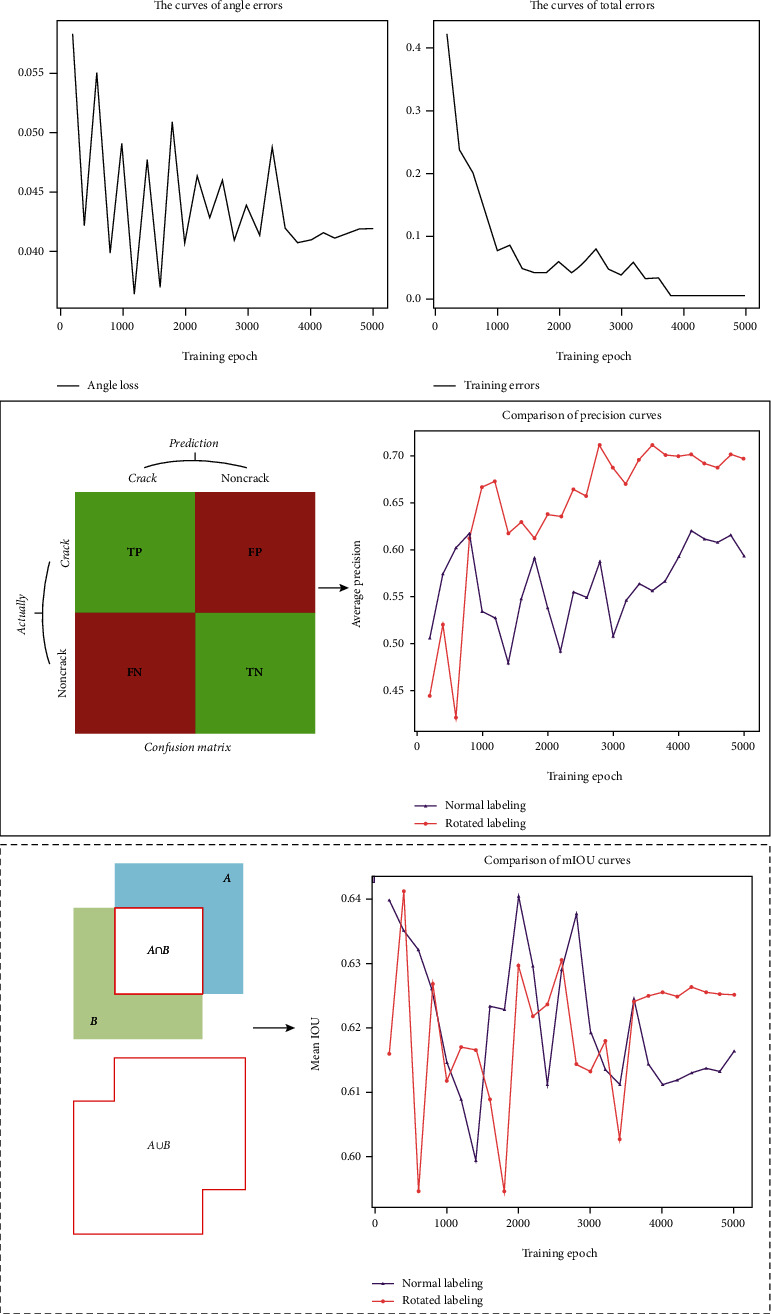
Qualitative analysis of experimental results. (a, b) The training loss of our approach. The precision curve represents the probability that the prediction is correct. The mIOU represents the average ratio of the intersection and union of the prediction box and the ground truth box.

The dataset with rotation annotation and conventional annotation was used to conduct experiments. Figures 7(a) and 7(b) are training loss curves with rotation annotations. The loss value will continue to decrease, as the number of training increases. Among them, the angle loss is accompanied by the attenuation of the learning rate, and the fluctuation range of the curve gradually decreases and tends to be stable. The comparison of accuracy curves of the two different methods is shown in the solid line box in Figure 7. It can be found that the accuracy of the rotating labeling method is significantly higher than that of the conventional labeling method, and its highest detection accuracy can reach 71.12%. The IOU curve of the rotating labeling method is not much different from the other method, and the highest mIOU can reach 64.13%, as shown by the dashed box in Figure 7. Since the rotating label frame has an angle loss, the IOU value fluctuates greatly due to the angle loss in the initial training stage. The mIOU also stabilizes when the angle loss stabilizes.

**Figure 8:**
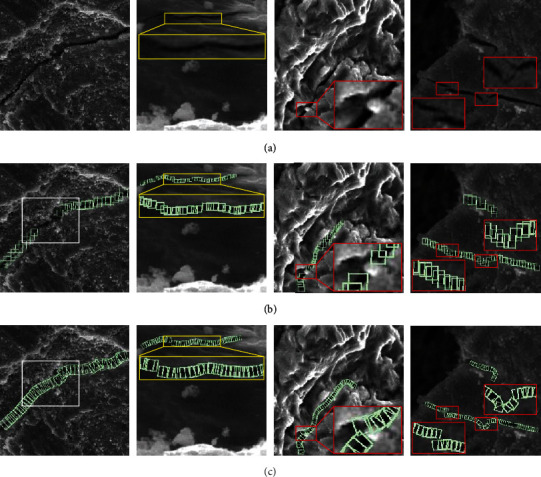
Qualitative comparison of different annotation methods. (a–c) Original image, conventional annotation, and rotation annotation, respectively.

The conventional labeling methods are prone to missed detections at local cracks, as shown by the white marks in the first column of Figure 8. Because each rotating bounding box is constrained by the angle, it can reduce the feature difference between different bounding boxes and improve the unity of crack local features. Therefore, the deep learning network model can obtain better quality high-dimensional feature maps and improve the overall detection performance of the model. As shown by the yellow marks in the second column of Figure 8, the bounding boxes returned by the conventional labeling methods have different sizes or messy positions. The regression error between the bounding boxes will be reduced, due to the angle constraint added in the training process of the rotation labeling method, the regression error between the bounding boxes will be reduced. Therefore, compared with the conventional labeling method, the detection result of the rotating labeling method is more compact, and the regression error is relatively less. There are local gaps in the cracks, as shown by the red marks in the third and fourth columns of Figure 8. With the help of the rotating bounding box, the regression state of the gap area can be adjusted to distinguish the local gap area of the crack.

#### 3.3.2. Comparison of Different Regressions

**Figure 9:**
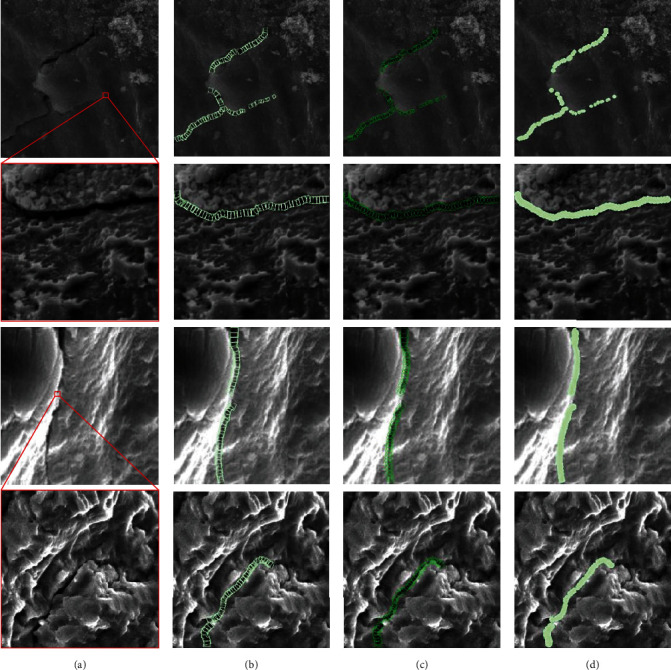
Qualitative comparison of different bounding boxes. (a–d) Original image, square bounding boxes, circle bounding boxes, and filled circle bounding boxes, respectively.

The imaging resolution change continuously. And the image background, illumination, and crack characteristics also change when scanning electron microscopy searches for damaged areas. In order to verify the feasibility of our method, Figure 9 shows the detection effects of comparing square bounding boxes, circle bounding boxes, and filled circle bounding boxes in SEM images with different resolutions and backgrounds. The background impurities are relatively small, and the surface topography is relatively smooth in low-resolution images. There are more fretting wear debris and wave-like fatigue stripes, and the morphology is more complex in high-resolution images. Comparing the four groups of test results, it is found that the regression area of circle regression is smaller and the edge area is smoother when identifying the crack trajectory. Since each bounding box identifies the part of the crack, a large number of dense bounding boxes are needed when expressing the overall characteristics of the cracks. The filled regression was used to replace the dense bounding box, which can make the detection result closer to the original shape of the cracks. There will be a small amount of confirmation of fine cracks when the background is dark, as shown in the first and second rows of Figure 9. The detection effect is better when the crack features are more obvious. The flat-shaped image has good detection performance when the background is bright, while a small amount of missing detection appears in the image with more obvious wave-like folds, as shown in the third and fourth rows of Figure 9. It can be seen from the second and fourth rows that deep learning performs well in images with complex shapes and can meet the detection requirements of scanning electron microscopes at different resolutions.

#### 3.3.3. Comparison between Different Methods

**Figure 10:**
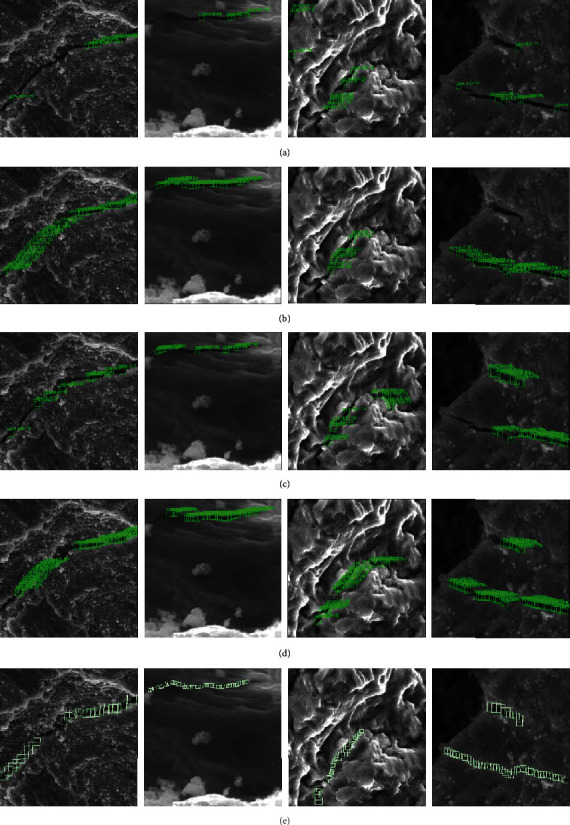
Qualitative comparison between different methods. (a–e) SSD, YOLO, RetinaNet, faster R-CNN, and our approach, respectively.

This paper is compared our approach with four different published methods which include SSD [49], RetinaNet [43], faster R-CNN [31], and YOLO [27], respectively. All methods used the same dataset and training parameters. The qualitative comparison results of five different methods are shown in Figure 10. The experimental result shows that the SSD algorithm has poor detection performance, and the faster R-CNN and our approach performed well. The first, second, and fourth column images are the microcrack images in the micromotion loss area. In the environment with more fretting wear debris, it can find that SSD and RetinaNet have many missed detection areas. As shown in the third column, there are many wave-like wrinkles in the fatigue fracture, which improves the detection difficulty of microcracks. For example, some background information is incorrectly identified using the SSD algorithm, and the other three public methods have different degrees of missed detection. But our approach still performed better.

## 4. Conclusion

A crack detection method with dense rotating bounding boxes based on the deep learning model was proposed to realize the intelligent recognition of microcracks in SEM images in this paper. Firstly, dense and continuous bounding boxes were used to mark along the microcrack growth trajectory to reduce the learning of background features by the deep learning model. Secondly, the rotation was used angle to reduce the difference in the local characteristics of the cracks in each bounding frame. Our model consists of a ResNet feature extractor and an anchorless frame-based CenterNet algorithm, which adds angle prediction to high-dimensional feature maps. The shape of the bounding box was modified and filled in color to optimize the detection effect of cracks in SEM images. The results show that the detection accuracy of our method reaches 71.12%, which can be used for SEM crack image detection with different magnifications. The regression state of rotation was used to successfully distinguish the local gap area of the crack. This paper successfully combined the deep learning method with the crack SEM image and found that it has great development potential in computer materials through experiments. In the future, we will consider how to embed deep learning algorithms into SEM to realize real-time, traceable, and more intelligent detection of microcracks.

## Figures and Tables

**Table 1:** Input features reused algorithm.

Layers	Input	Filters	Kernel	Output
0:Conv	512 × 512 × 3	64	7 × 7	256 × 256 × 64
1:Maxpool	256 × 256 × 64	64	3 × 3	128 × 128 × 64
2:Block × 3	128 × 128 × 64			128 × 128 × 256
3:Conv	128 × 128 × 128	128	1 × 1	64 × 64 × 128
4:Block × 4	64 × 64 × 128			64 × 64 × 512
5:Conv	64 × 64 × 512	256	1 × 1	32 × 32 × 256
6:Block × 6	32 × 32 × 256			32 × 32 × 1024
7:Conv	32 × 32 × 1024	512	1 × 1	16 × 16 × 512
8:Block × 3	16 × 16 × 512			16 × 16 × 2048
9:Deconv	16 × 16 × 2048	256	3 × 3	16 × 16 × 256
10:Prediction	16 × 16 × 256			512 × 512 × 3

Layers: the functional layers in the model; Block: a residual block;, input and output represent the shape of tensor; Kernel: the size of convolution kernel; and Filters: the number of convolution kernels.

## Data Availability

Data can be available upon request.

## Additional Points

figs/1.jpg Lun Zhao received the Ph.D. degree in Mechanical Design and Theory from Kunming University of Science and Technology, Kunming, China, in 2018. He is currently worked as a post-doctoral researcher in Shenzhen Institutes of Advanced Technology, Chinese Academy of Sciences, Shenzhen, China. His research interests cover new technology of sheet joining, additive manufacturing and machine learning. figs/2.png Yun Long Pan received his B.S. degree from City College of Dongguan University of Science and Technology in 2019. Now, he is pursuing his M.S degree in Kunming University of Science and Technology. His main research interests include machine vision detection and deep learning algorithms. figs/3.jpg Sen Wang received the Ph.D. degree in the direction of mechanical and electronic engineering from Kunming University of Science and Technology, Kunming, China, in 2017. He is currently a lecturer with the Faculty of Mechanical and Electrical Engineering, Kunming University of Science and Technology. His research interests concentrate on image processing, computer vision and deep learning. figs/4.jpg Liang Zhang received the Ph.D. degree in Materials Science and Engineering from Haerbin Institute of Technology, Haerbin, China, in 2013. He is an Associate Professor in Shenzhen Polytechnic, Shenzhen, China. His research interests include additive manufacturing and welding. figs/5.jpg Md Shafiqul Islam received his Ph.D. degree in mechanical engineering from Blekinge Institute of Technology, Blekinge, Sweden, in 2019. He is currently working as a postdoctoral researcher at the Faculty of Mechanical Engineering, Blekinge Institute of Technology. His research interests cover fracture mechanics, constitutive modelling, and machine learning.
